# Bibliometric and visual analysis of diabetes mellitus and pyroptosis from 2011 to 2022

**DOI:** 10.1186/s40001-023-01175-7

**Published:** 2023-07-13

**Authors:** Xiaodong Li, Xiaojuan Su, Fenglin Xia, Jing Qiu, Jiaqi Zhang, Haiyan Wu, Xuejun Xie, Mingchao Xu

**Affiliations:** 1 The First Affiliated Hospital of Guizhou University of Chinese Medicine, Guiyang, 550000 China; 2 Chengdu University of Traditional Chinese Medicine, Chengdu, 610075 China; 3 Hospital of Chengdu University of Traditional Chinese Medicine, Chengdu, 610072 China; 4 Traditional Chinese Medicine Hospital of Meishan, Meishan, 620010 China

**Keywords:** Diabetes mellitus, Diabetic complications, Pyroptosis, Bibliometrics

## Abstract

**Objective:**

To visualize and analyze the published literature on diabetes mellitus and pyroptosis based on a bibliometric approach, so as to provide a comprehensive picture of the hot research directions and dynamic progress in this field.

**Methods:**

This study was based on the web of science core collection database to conduct a comprehensive search of the published literature in the field of diabetes mellitus and Pyroptosis from January 1985 to August 2022, including the published research literature in this field, as well as a visual analysis of the number of citations, year of publication, journal, author, research institution, country, and research topic.

**Results:**

A total of 139 literature on research related to diabetes mellitus and cellular scorch from 2011 to 2022 were retrieved, with a total of 3009 citations and a maximum of 255 citations for a single article, which had a first author Schmid-Burgk, JL The first author of this article is from Germany; among 20 publishing countries, China leads with 100 articles; among 222 publishing institutions, Harbin Medical University leads with 18 articles and 184 citations; among 980 authors, Chen, X from China tops the list of high-impact authors with 5 articles and 29 citations. Among the 98 journals, "CELL DEATH DISEASE" ranked first in both volume and high-impact journals with 4 articles and 29 citations. Among 349 keywords, "pyroptosis" ranked first with a cumulative frequency of 65 times. The cluster analysis was divided into three categories, chronic complications of diabetes mellitus and pyroptosis (67 articles), diabetes mellitus and pyroptosis (60 articles), and diabetes mellitus combined with other diseases and pyroptosis (12 articles), and the number of articles related to diabetes mellitus and its chronic complications increased rapidly from 2019, among which, diabetic cardiomyopathy (27 articles) had the highest number of articles.

**Conclusions:**

Based on a comprehensive analysis of published literature in the field of diabetes mellitus and pyroptosis from 2011 to 2022, this study achieved a visual analysis of studies with significant and outstanding contributions to the field, thus framing a picture showing the development and changes in the field. At the same time, this study provides research information and direction for clinicians and investigators to conduct diabetes mellitus and pyroptosis-related research in the future.

## Introduction

Diabetes mellitus is a complex metabolic disease characterized by systemic dysregulation of glucolipid metabolism and chronic, low-grade inflammation caused by hyperglycemia [1]. The 10th edition of the IDF Diabetes mellitus Atlas suggests that more than 1 in 10 adults worldwide will have diabetes mellitus in 2021, and that the number of diabetes mellitus cases will continue to increase rapidly in the future, with the increasing incidence of diabetes mellitus due to aging and urbanization. Pyroptosis is a pro-inflammatory mode of programmed cell death in which the activation of pore-forming protein-D (GSDMD) by NLPR3 inflammatory vesicles induces the formation of pores in the cytosol, leading to cell swelling and rapid lysis of cell contents and the release of several pro-inflammatory mediators such as interleukin-1β (IL-1β) and interleukin-18 (IL-18) [2]. It has now been shown that scorch death is involved in the inflammatory response and can promote the progression of diabetes mellitus and its multiple complications [3, 4]. Some molecular proteins targeting pyroptosis and related signaling pathways may be a new potential target for the management and treatment of diabetes mellitus and its complications. In this study, a comprehensive quantitative and visual analysis of the literature related to diabetes mellitus and pyroptosis research was carried out through bibliometric analysis with the help of VOSviewer and other related professional literature analysis software. In addition, for clinicians and scholars studying diabetes, its complications and pyroptosis, the results of this study will not only provide information on the hot spots and dynamic progress of research in this field, but also provide important research directions.

## Information and methodology

Data were from the Web of science database (http: //web of science.com); search method, a combination of subject and free word search; time span from 1985 to 2022, type of literature is thesis, language is English, and the search time was set to August 13, 2022.

### The search formula is as follows

#1 TOPIC:(diabetes mellitus) OR TOPIC:(diabetes) OR TOPIC:(diabetic) OR TOPIC:(glycosuria) OR TOPIC:(glycuresis) OR TOPIC:(Diabetes Mellitus, Type 1) OR TOPIC:(T1DM) OR TOPIC:( Diabetes Mellitus, Type 2) OR TOPIC:(T2DM).

#2 TOPIC:( Pyroptosis) OR TOPIC:(Pyroptoses) OR TOPIC:(Cell Deaths, Pyroptotic) OR TOPIC:(Cell Deaths, Pyroptotic) OR TOPIC:(Deaths, Pyroptotic Cell) OR TOPIC:(Deaths, Pyroptotic Cell) OR TOPIC:(Deaths, Pyroptotic Cell) OR TOPIC:(Pyroptotic Cell Deaths) OR TOPIC:(Caspase-1 Dependent Cell Death) OR TOPIC:(Caspase 1 Dependent Cell Death) OR TOPIC:(Inflammatory Apoptosis) OR TOPIC:(Apoptoses, Inflammatory) OR TOPIC:(Apoptosis, Inflammatory) OR TOPIC:(Inflammatory Apoptoses).

#3 #1 AND #2, Indexes = SCI-EXPANDED, SSCI, Timespan = 1985–2022.

### Inclusion and exclusion criteria

1. Inclusion criteria: published research-based English-language thesis literature relevant to the study of diabetes mellitus and pyroptosis was met.
2. Exclusion criteria: reviews, narrative reviews, guidelines, consensus, and literature with incomplete information.

### Methods

The literature type was selected as thesis and after that the search page was updated to get the updated search results. After selecting all the results, the record content was selected as full record for export and the export format was txt format. Statistical collation and visual analysis of the included literature data package was performed through VOSviewer 1.6.18 freeware and Microsoft Excel 2019 (no license required) [5]. The main information extracted included authors, year of publication, journal of publication, number of citations, scientific institution, country or region, keywords and other important literature data, and the research topics were identified through the titles, keywords and abstracts of the literature and, if necessary, through full-text reading analysis. All the search and inclusion process was done independently by two researchers, and the literature or data with disagreement was then independently confirmed by a third researcher.

## Results

### Distribution of literature publication time

**Fig. 1:**
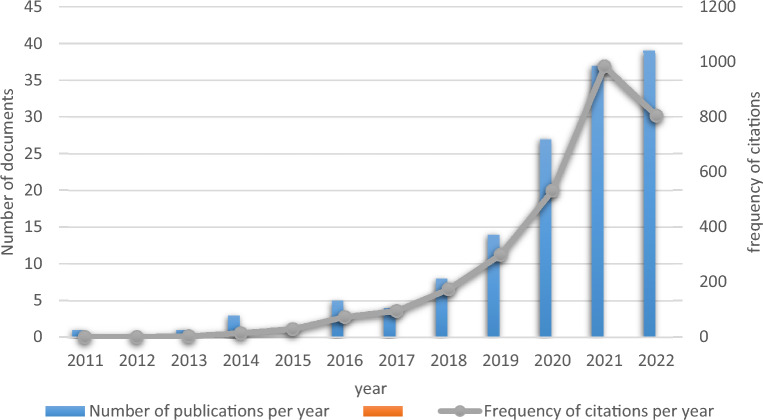
Infographic of the number of publications and frequency of citations in the field of diabetes mellitus and pyroptosis over the years

The literature search revealed that there was no published literature on diabetes mellitus and pyroptosis-related studies between 1985 and 2011. From 2011 to 2022, a total of 139 publications were searched in this field, with an average of 11.6 publications per year, of which no publications were retrieved in 2012 and 2015, and the number of publications increased rapidly from 2018 onwards, with 39 publications in 2022 as of August (Fig. 1).

### Number of citations per year of published literature

From 2011 to 2022, 139 published literature were cited 3009 times, 2877 times after removing self-citations, with a mean H-index of 27 (Fig. 1). The top citation frequency for a single published literature was by an author from Germany Schmid-Burgk, JL [6] In addition, 8 of the top 10 cited articles were from China, as shown below (Table 1).

**Table 1:** Table of information on the top 10 highly cited publications in the field of diabetes mellitus and pyroptosis

First author	Annual	National	Periodical	Total number of citations	Average
Schmid-Burgk, JL [6]	2016	German	Journal of Biological Chemistry	255	36.43
Li, X [7]	2014	Sino	Cell Death & Disease	190	21.11
Qiu,Z [8]	2017	Sino	Oxidative Medicine and Cellular Longevity	177	29.50
Luo, BB [9]	2014	Sino	Plos One	166	18.44
Li, X [10]	2017	Sino	Experimental Cell Research	159	26.50
Giordano, A [11]	2013	Italy	Journal of Lipid Research	154	15.40
Yang, F [12]	2019	Sino	International Journal of Biological Sciences	122	30.50
Yang, F [13]	2018	Sino	Cell Death & Disease	117	23.40
Qiu,Z [14]	2019	Sino	Journal of Diabetes Research	109	27.25
Yang, F [15]	2018	Sino	Cellular Physiology and Biochemistry	90	18

### Number of publications in different countries/regions

**Fig. 2:**
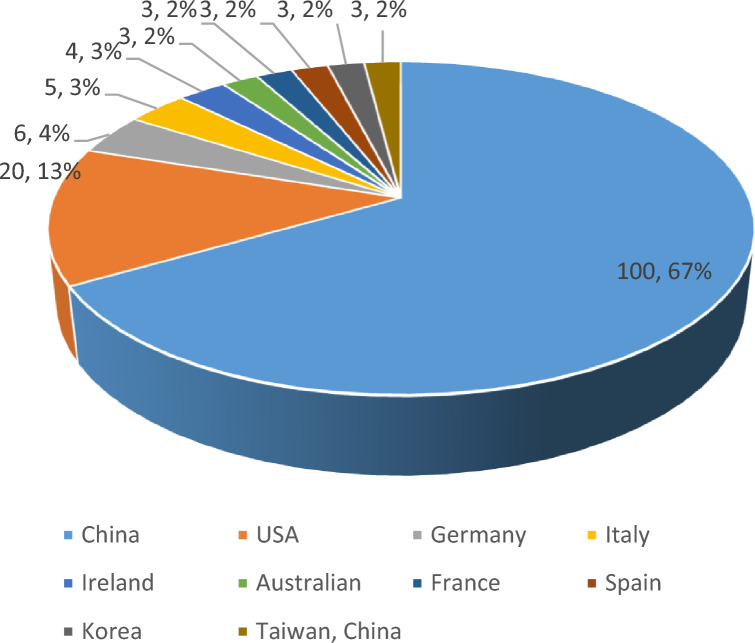
Top 10 countries/regions in terms of cumulative number of publications

**Fig. 3:**
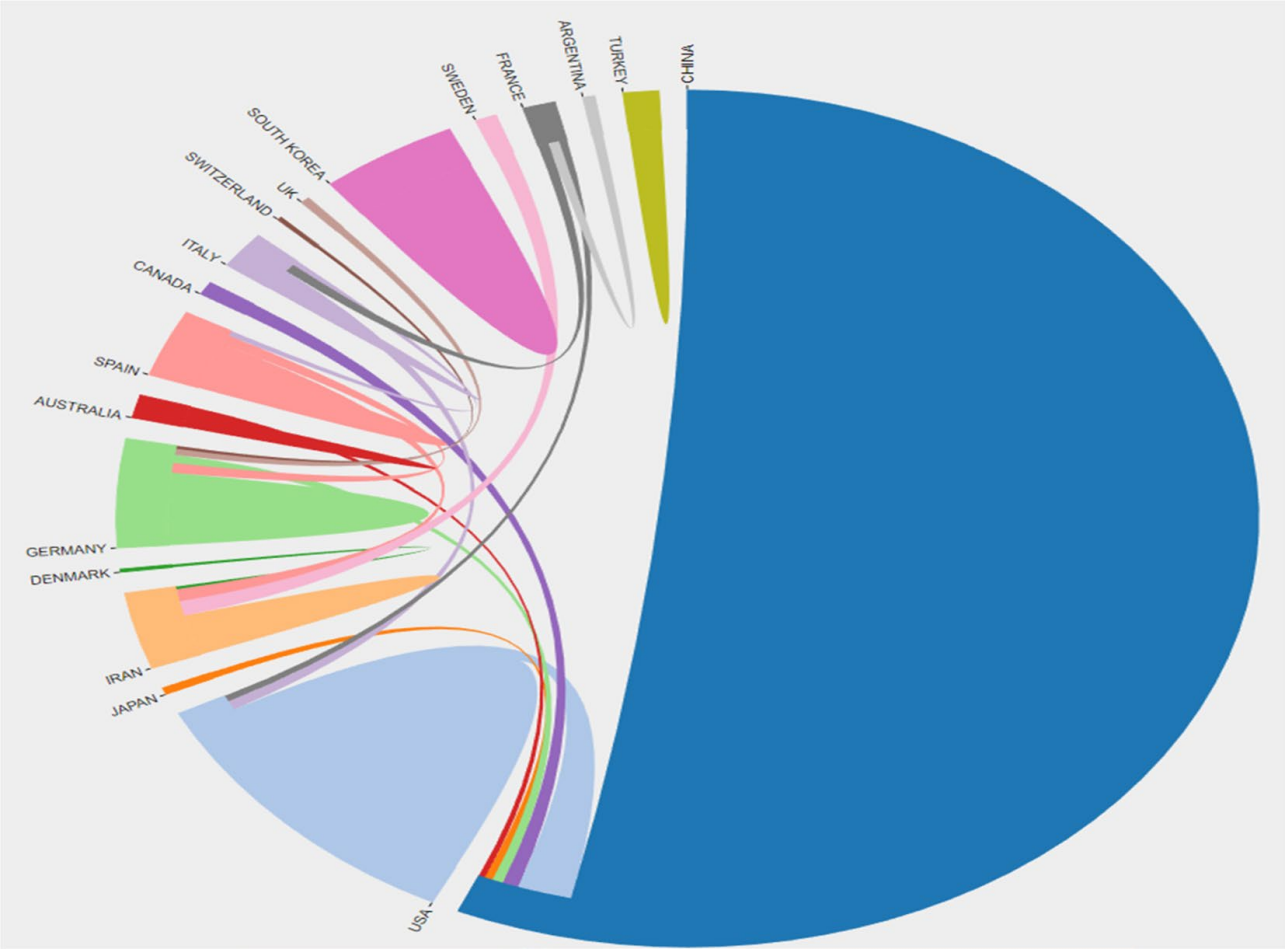
Distribution of collaborative relationships in published literature by country/region

4. In 2011–2022, a total of 20 countries/regions published literature related to diabetes mellitus and pyroptosis research, and China has accumulated 100 publications so far, accounting for 67%, ranking first, followed by the United States (20, 13%), Germany (6, 4%), Italy (5, 3%). The volume of publications from other countries is shown in Fig. 2. At the same time, there is a close cooperation between these publishing countries/regions, as shown in Fig. 3.

### The number of publications and citations of different research institutions

**Fig. 4:**
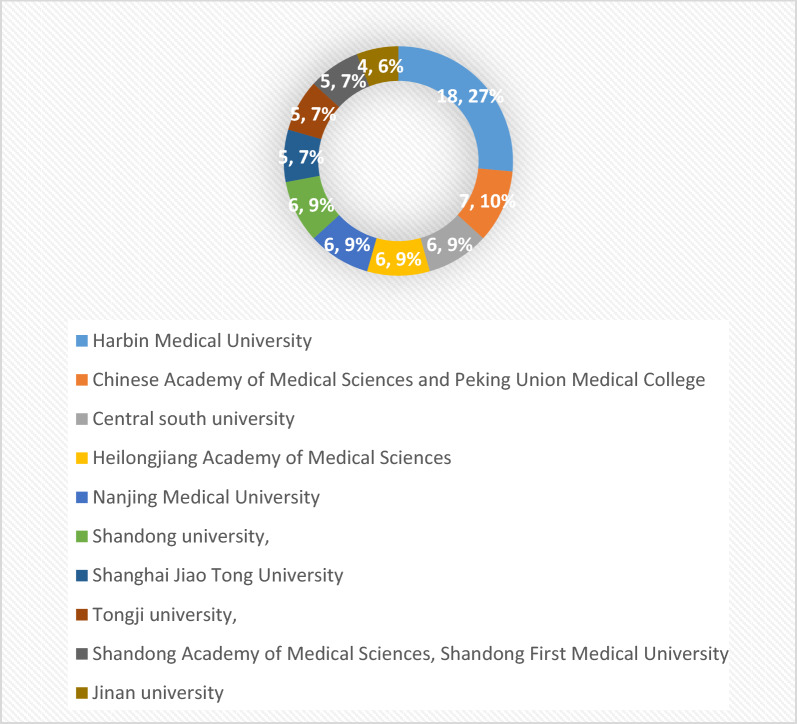
Top 10 scientific institutions in the field of diabetes mellitus and pyroptosis in terms of number of publications

**Fig. 5:**
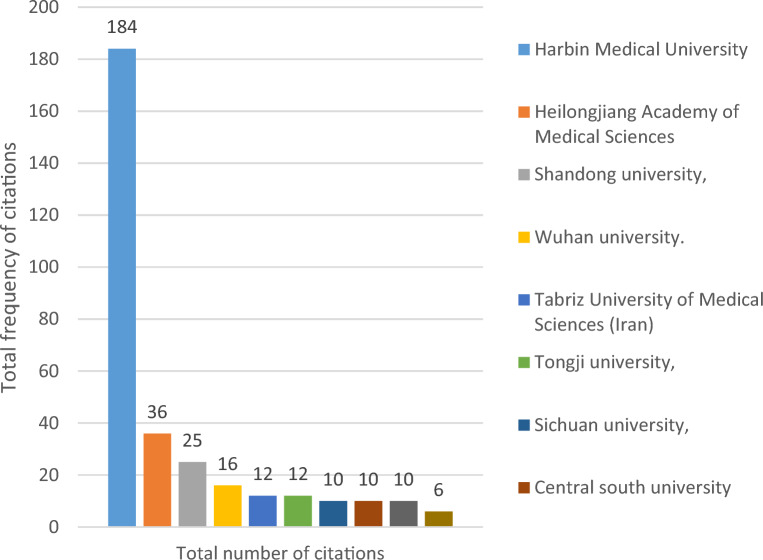
Top 10 highly cited scientific institutions in published literature in the field of diabetes mellitus and pyroptosis

**Fig. 6:**
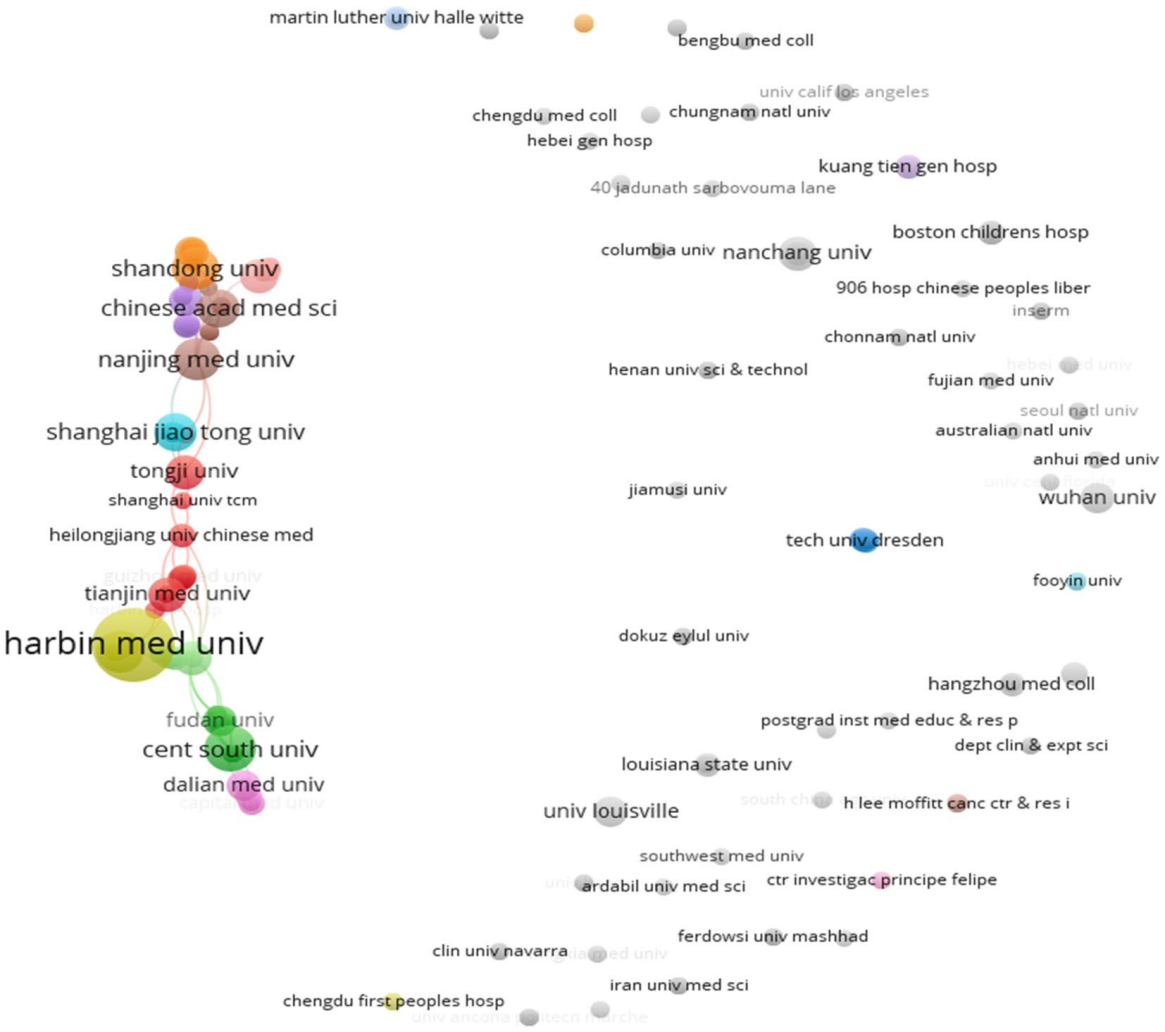
Collaboration of scientific institutions publishing literature in the field of diabetes mellitus and pyroptosis

The 139 publications from 2011 to 2022 involved 222 relevant universities and other research institutions, among which, Harbin Medical University ranked first with 18 publications; Peking Union Medical College of the Chinese Academy of Medical Sciences ranked second with 7 publications; the top 10 research institutions with the most publications were all from China (Fig. 4). In addition, from the analysis of the citations of the published literature of research institutions, Harbin Medical University is the most influential research institution in this field with 184 total citations, followed by domestic and foreign university research institutions such as Heilongjiang Academy of Medical Sciences (36 total citations) and Shandong University (25 total citations) (Fig. 5), as shown in Fig. 6, there are also close academic exchanges and cooperation among these research institutions relationship.

### The number of publications and citations by different authors

**Fig. 7:**
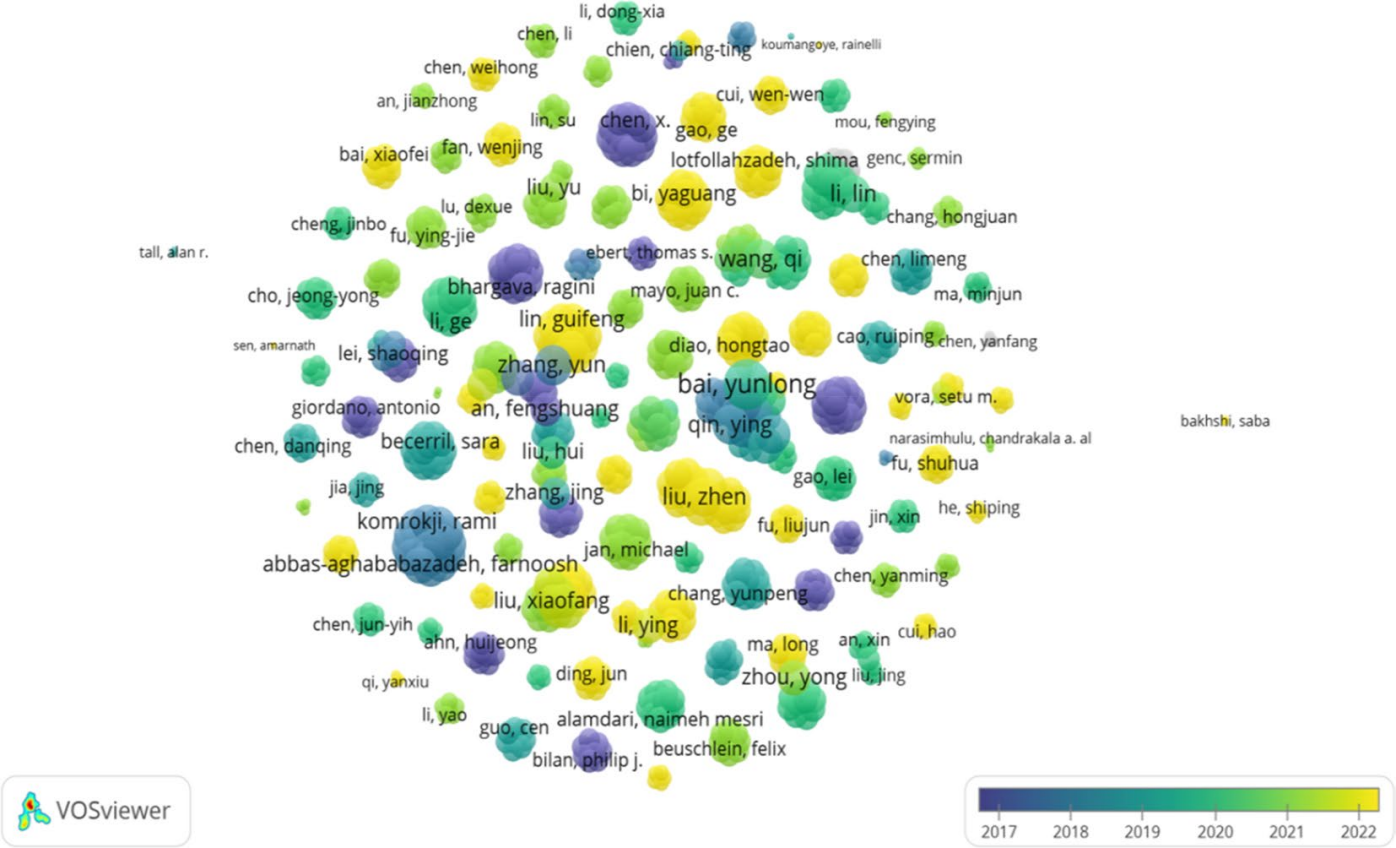
Distribution of collaborative relationships between authors of published literature on diabetes mellitus and pyroptosis studies

A total of 980 authors collaborated with each other to publish 139 papers related to the field of diabetes mellitus and pyroptosis from 2011 to 2022, and the top 10 influential authors are shown in Table 2, with author Chen, X (5 publications, 29 total citations) ranking first in terms of influence, followed by Wang, Y Q, Che, H, and Wang, L H (4 publications, 29 total citations). In addition, the collaboration between different authors is shown in Fig. 7.

**Table 2:** Top 10 authors with high citations in published literature on diabetes mellitus and pyroptosis research

Author name	Total literature	Total number of citations	Average number of citations	Number of operations	Number of communications
Chen, X	5	29	5.80	0	0
Wang, Y Q	4	29	7.25	0	0
Che, H	4	29	7.25	1	0
Wang, L H	4	29	7.25	0	3
Yang, F	3	28	9.33	3	0
Qin, Y	4	28	7.00	0	0
Lv, J	3	28	9.33	0	0
Bai, Y L	6	28	4.67	1	1
Zhang, Y	6	28	4.67	0	1
Li, X	4	23	5.75	2	0

### Number of literature published in different journals

139 papers included in this study from 2011 to 2022 were published in 98 different journals and magazines, respectively. Based on the data of journal volume and highly cited information, "CELL DEATH DISEASE" is the top journal in both volume and impact in the field of diabetes mellitus and pyroptosis, with Chinese Academy of Sciences (CAS) region I, impact factor 9.685, with an H-index of 59. In addition, the top 10 journals in terms of number of articles and high citations are shown in Tables 3 and 4, respectively.

**Table 3:** Information on the top 10 journals in terms of cumulative number of publications in diabetes mellitus and pyroptosis research

Journal name	Number of articles issued	Impact factor	CAS division	H-index
Cell Death Disease	4	9.685	First	59
Biochemical and Biophysical Research Communications	4	3.322	Third	43
Frontiers in Pharmacology	4	5.988	Second	57
International Immunopharmacology	4	5.714	Second	40
Life Sciences	4	6.780	Third	51
Oxidative Medicine and Cellular Longevity	4	7.310	Second	49
Bioengineered	3	6.832	Quadrant	22
Biomedicine Pharmacotherapy	3	7.419	Second	59
Journal of Diabetes Investigation	3	3.681	Third	22
Journal of Diabetes Research	3	4.061	Third	24

*CAS* Chinese Academy of Sciences

**Table 4:** Information on the top 10 highly cited journals for published literature on diabetes mellitus and pyroptosis research

Journal name	Number of articles issued	Total number of citations	Average number of citations	CAS division
Cell Death Disease	4	29	7.25	First
Plos One	2	14	7.00	Third
Oxidative Medicine and Cellular Longevity	4	11	2.75	Second
Cellular Physiology and Biochemistry	1	8	8.00	Uncatalogued
International Journal of Biological Sciences	2	7	3.50	Second
Biochemical and Biophysical Research Communications	4	6	1.50	Third
Journal of Diabetes Research	3	6	2.00	Third
Experimental Cell Research	1	6	6.00	Third
Journal of Lipid Research	2	5	2.50	Second
Biomed Research International	1	3	3.00	Third

### Analysis of research hotspots

**Fig. 8:**
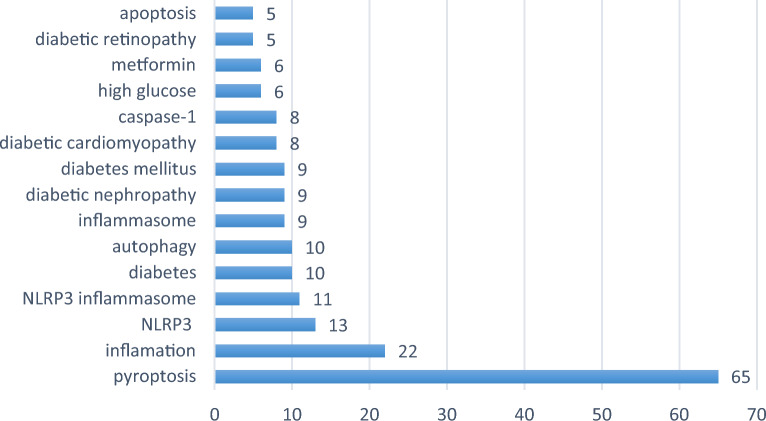
Top 15 keywords in cumulative frequency of published literature on diabetes mellitus and pyroptosis research

**Fig. 9:**
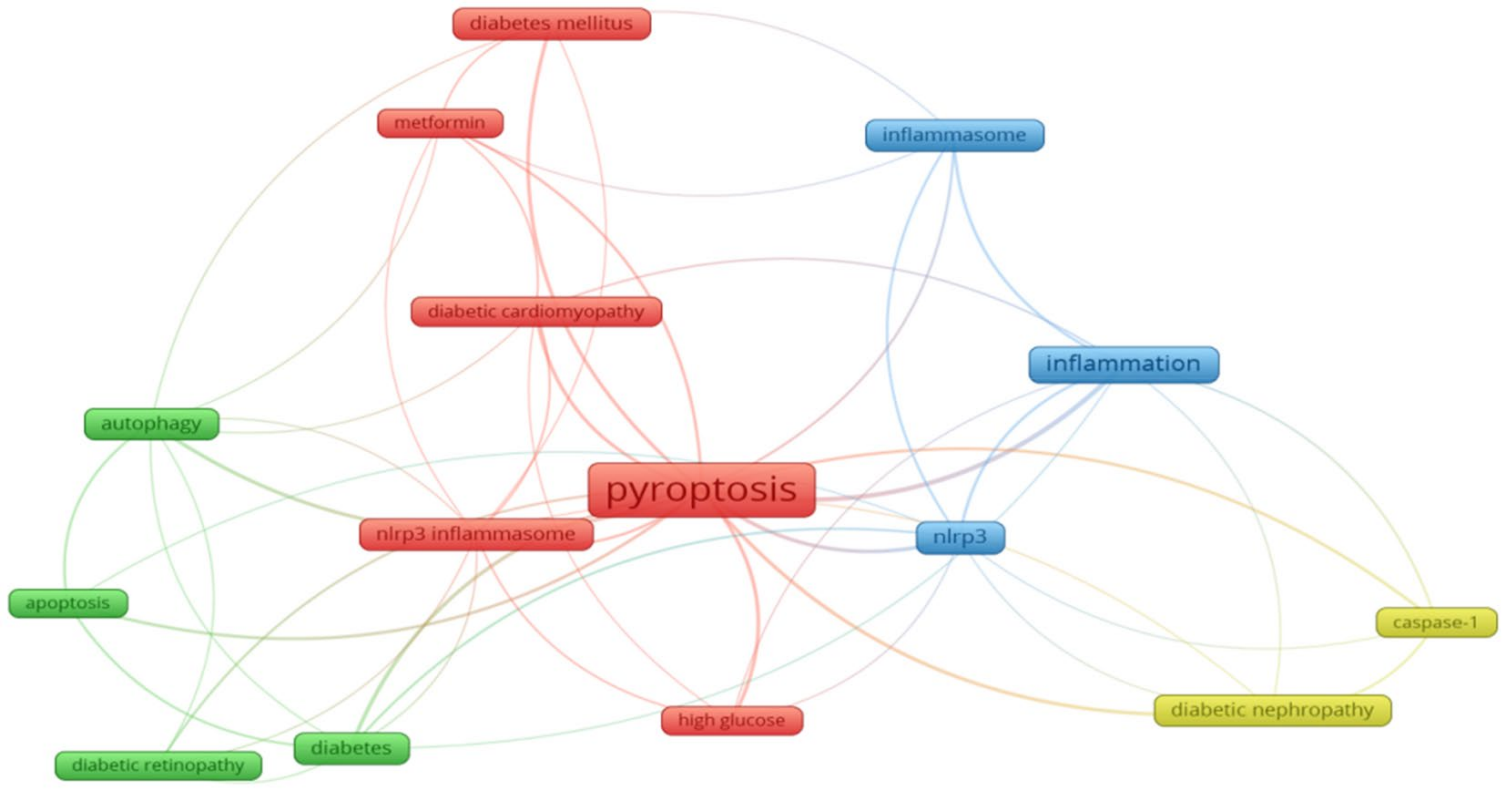
Cluster analysis network relationship diagram of high-frequency keywords in published literature on diabetes mellitus and pyroptosis research

**Fig. 10:**
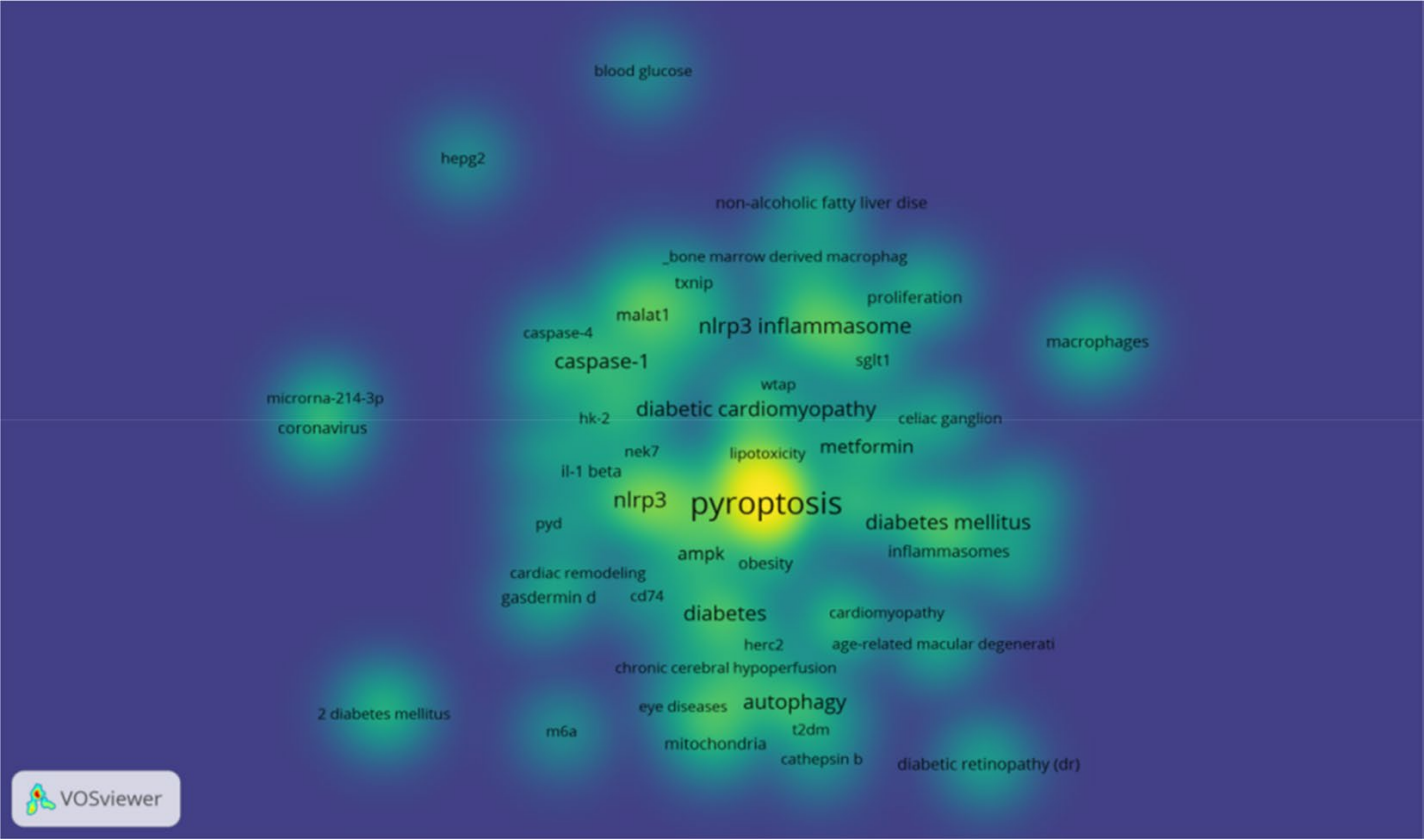
Distribution of keyword density in published literature on diabetes mellitus and pyroptosis studies

The frequency of keywords in the published literature can be used to analyze the current hotspots in the research field. From 139 papers published in 2011-2022, 349 keywords can be extracted, the first keyword "cell death" has a cumulative frequency of 65, the second and third keywords are "inflammation" and "NLRP3", respectively. And the other top 15 high-frequency keywords are shown in Fig. 8. In addition, as shown in Fig. 9, the clustering network analysis of high-frequency keywords by VOSviewer revealed that the key proteins of pyroptosis, "NLRP3", "inflammatory vesicles", "NLRP3 inflammatory vesicles", and "caspase-1" centered on "pyroptosis", while "autophagy", and "apoptosis" were involved. "The only relevant therapeutic drug is "metformin" among the high-frequency keywords, which is involved in the development of hyperglycemia, diabetes mellitus and its chronic complications diabetic retinopathy (DR), diabetic nephropathy (DN) and diabetic cardiomyopathy (DCM). The density distribution of keywords in the published literature in the field of diabetes mellitus and pyroptosis is shown in Fig. 10.

### Analysis of the dynamics of research themes

**Fig. 11:**
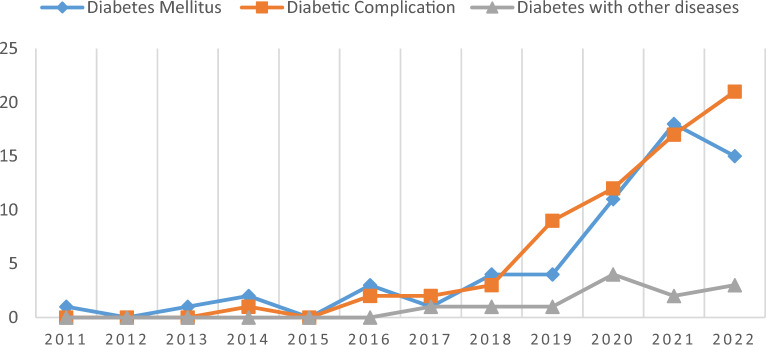
Trends in diabetes mellitus and pyroptosis research themes, 2011–2022

By thematically locating and classifying the literature included in this study, it was found that the largest number of literature (67) studied the association of pyroptosis with diabetic complications, including DCM (27), DN (17) and DR (10), followed by literature (60) that studied the association of pyroptosis with the pathogenesis and therapeutic targets of diabetes, and finally, literature related to diabetes mellitus combined with lastly, the literature related to other diseases (12). From the analysis of the timeline of literature publication, 2 papers published in 2011 and 2013 were on the topic of diabetes mellitus and pyroptosis, 1 paper was published in 2014 on the topic of diabetic complications, no literature published in 2012 and 2015 on the above related topics were inquired, 1 paper was published in 2017 on the topic of diabetes mellitus combined with other diseases, and before 2019. The number of publications on all 3 of the above topics was low, and then the number of publications on diabetes mellitus and its complications with pyroptosis studies increased year by year, especially the rate of publications on diabetes complications with pyroptosis studies increased faster (Fig. 11).

**Fig. 12:**
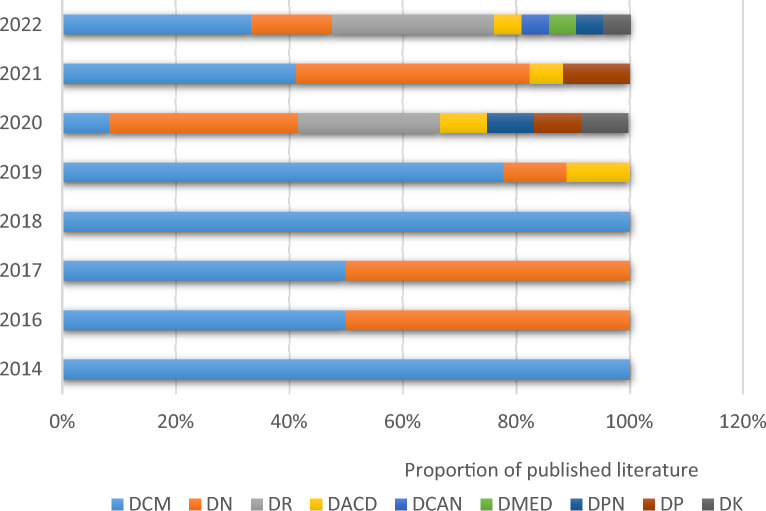
Proportional distribution of the number of publications per year in diabetes complications and pyroptosis studies, 2014–2022 (DCM: diabetic cardiomyopathy; DN: diabetic nephropathy; DR: diabetic retinopathy; DACD: diabetes-related cognitive decline; DCAN: diabetic cardiac autonomic neuropathy; DMED: diabetes mellitus-induced erectile dysfunction; DPN: diabetic peripheral neuropathy; DP: diabetic periodontal disease; DK: diabetic keratopathy)

The first literature studying diabetic cardiomyopathy and pyroptosis was published in 2014, which was also the first literature related to diabetic complications and pyroptosis until 2019, the number of published literature studying diabetic cardiomyopathy ranked first, followed by diabetic nephropathy and diabetic cognitive dysfunction. From 2020, the number of published literature on diabetic complications and pyroptosis studies has increased rapidly, and there has been a gradual increase in published literature on different types of diabetic complications, such as diabetic retinopathy, diabetic keratopathy, and diabetic periodontitis. From 2014 to the present, diabetic cardiomyopathy and diabetic nephropathy have ranked first in terms of the percentage of published literature per year, in addition to 2020 The proportion of the annual number of published literature for diabetic retinopathy has increased rapidly and has ranked second by 2022 (Fig. 12).

## Discussion

During this past approximately 12 years, diabetes mellitus and pyroptosis have made progress in areas related to research on pathogenesis, signaling pathways, and therapeutic targets, which have far-reaching implications for future diabetes-related basic research and clinical treatment strategies. This study provides a comprehensive, quantitative and visual analysis of 139 papers related to diabetes mellitus and pyroptosis published in the past 12 years based on bibliometrics. With the rapid development of the new era and the increasing trend of globalization, such as scientific and technological research, the research articles included in this study were completed by researchers collaborating with each other among research institutions in different countries or regions. From the analysis of the timeline, the number of published literature on diabetes mellitus and pyroptosis has been increasing year by year, especially in the past three years, which indicates that although the research on diabetes mellitus and pyroptosis started late, it has developed more rapidly and received more and more attention in recent years. These data show that Chinese universities have made outstanding contributions in this field, and they also show that universities in China and abroad are important institutions to promote the progress of research in this field. In addition, the quality level of Chinese scholars' research in this field has been recognized internationally.

## Diabetes mellitus and pyroptosis

The results of high-frequency keyword analysis in this study show the direction of diabetes mellitus and pyroptosis research in recent years, mainly around the link between the specific mechanisms by which pyroptosis occurs as an inflammatory response and the pathogenesis and potential therapeutic targets of diabetes. The classical pyroptosis pathway consists of activation of caspase 1 by activated NLPR3 inflammatory vesicles to cleave GSDMD and then form membrane pores in the cell membrane, followed by the efflux of intracellular contents and release of a large number of inflammatory factors such as IL-1β and IL-18 [16, 17]. The non-classical pyroptosis pathway is a pathway predicated on the activation of Caspase-4/5/11 [18]. The important pathological features of diabetes mellitus are insulin resistance and pancreatic β-cell destruction leading to impaired insulin secretion, and previous studies have shown a close relationship between the pathogenesis of diabetes mellitus and the chronic inflammatory response [19] Carlos et al. [20] demonstrated that mitochondrial DNA activates NLPR3 inflammatory vesicles and releases the pro-inflammatory factor IL-1β to induce type 1 diabetes. In a hyperglycemic environment, reactive oxygen species (ROS) activate NLRP3 inflammatory vesicles in pancreatic β-cells, promoting IL-1β secretion leading to insulin secretion dysfunction, promoting obesity and insulin resistance, and ultimately inducing type 2 diabetes mellitus raw [21]. Palmitic acid (PA) and lipopolysaccharide (LPS)-treated HepG2 cells induce NLRP3 inflammatory vesicles to activate caspase-1 and produce excessive pro-inflammatory cytokines such as IL-1β, IL-18 and TNF-α, impairing the action of insulin receptors, thereby blocking downstream signaling pathways and exacerbating insulin resistance in HepG2 cells. Furthermore, human umbilical cord-derived mesenchymal stem cells (UC-MSCs) co-cultured with HepG2 could effectively alleviate PA and LPS-induced insulin resistance by blocking NLRP3 inflammatory vesicle activation and inflammatory factors. In addition, downregulation of NLRP3 or IL-1β expression partially ameliorated impaired insulin signaling in UC-MSCs. Similarly, UC-MSCs significantly improved hyperglycemia and reduced inflammatory factor activity in type 2 diabetic rats, thereby improving insulin resistance [22]. Hypoxia and islet inflammation are involved in pancreatic β-cell failure in type 2 diabetes, and a recent study [23] showed that activation of NLRP3 inflammatory vesicles in mouse insulinoma cells under hypoxic conditions leads to an inflammatory response and death of pancreatic β cells and upregulation of ROS and thioredoxin-interacting protein (TXNIP), and finally, TXNIP knockdown experiments in mouse insulinoma cells pretreated with the ROS inhibitor N-acetylcysteine (NAC) revealed that activation of ROS/TXNIP/NLRP3 axis is involved in hypoxia-induced inflammatory responses and cell death in pancreatic β-cells [23]. Tripartite motif‐containing (TRIM) family proteins as regulators are involved in both autophagy and pyroptosis, while they play critical roles in diabetes mellitus and its complications [24].TRIM27 [25], TRIM32 [26], TRIM7 [27], TRIM72 [28], TRIM13 [29] and TRIM63 [30] acting as regulatory proteins are involved in the development of DM as well as diabetic complications. The above studies suggest that pyroptosis is emerging as a new research perspective in the pathogenesis and treatment of diabetes. However, there are fewer studies on diabetes mellitus and pyroptosis. Whether scorched cell death of pancreatic cells leads to disorders of insulin secretion, especially in trials related to pancreatic β-cell function, needs to be further investigated.

The results of the progress of this research theme show that the number of published studies on diabetic complications and pyroptosis is increasing year by year, especially the study of DCM, DN and DR, the three major chronic complications of diabetes mellitus in pyroptosis, is gradually becoming a current hot spot.

## Diabetic cardiomyopathy and pyroptosis

DCM is a chronic complication of diabetes, characterized by myocardial fibrosis, left ventricular hypertrophy, impaired systolic and diastolic function, which can lead to heart failure and is a key cause of death in diabetic patients [31]. Chronic inflammation and fibrosis of the myocardium are important pathological changes in DCM [32]. An increasing number of studies have shown that activation of NLRP3 inflammatory vesicles in cardiomyocytes is involved in the development of DCM [33]. The Chinese medicine gibberellin can reduce the activation of NLRP3 inflammatory vesicles by inhibiting the production of ROS, thus ameliorating the high glucose-induced myocardial injury [34]. Compound Pearl Lipid Regulating Formula is an herbal preparation used clinically to treat disorders of glucolipid metabolism, and a recent study found that it could inhibit DCM by suppressing cardiac lipotoxicity-induced oxidative stress and NLRP3 inflammatory vesicle activation [35]. Santosh K et al [36] found that hyperglycemia upregulates oxidative stress-induced cell death via apoptosis and pyroptosis in human Cardiac Stem Cells (hCSCs), which is mediated by MMP9. Absence/knockdown of MMP9 improves viability of hCSCs by decreasing oxidative stress and suppressing downstream cell death signaling via apoptosis and pyroptosis. These results suggest that MMP9 may be an effective molecular target to interfere with the progression of DCM. More progress has been made in the study of signaling pathways related to DCM pyroptosis, including TLR4/NF-kB/NLRP3 inflammatory vesicle signaling pathway [37, 38], AMPK/ROS/TXNIP/NLRP3 inflammatory vesicle signaling pathway [39], AMPK/SIRT1/Nrf2/HO-1/NF-kB inflammatory vesicle signaling pathway [40, 41] and the FoxO3a/ARC/caspase-1 signaling pathway [7]; Yang F et al [12] demonstrated that metformin could inhibit NLRP3 inflammatory vesicles via the AMPK/mTOR/autophagy pathway, and also that metformin could block the expression of GSDMD-N, thereby slowing down the damage caused by pyroptosis in DCM. Recent study [42] found that hydrogen inhalation reduces the scorched death inflammatory response and ameliorates fibrosis by inhibiting the AMPK/mTOR/NLRP3 signaling pathway and inhibits the TGF-131/Smad signaling pathway, and that hydrogen in combination with metformin exhibits more effective cardioprotection in the treatment of DCM. In recent years, an increasing number of studies have shown that microRNAs (miRNAs), long-stranded non-coding RNAs (lncRNAs) and circular RNAs (circRNAs) may be biomarkers or potential therapeutic targets for DCM. LncRNA Kcnq1ot1 is involved in many cardiovascular diseases, and studies have demonstrated that silencing LncRNA Kcnq1ot1 inhibits miR -214-3p/caspase-1 pathway alleviates cardiomyocyte scorching in DCM mouse models and improves cardiac function and fibrosis [15]. In addition, caspase-1-related circular RNA (CACR) was increased in both high-glucose-treated cardiomyocytes and serum from diabetic patients, and CACR, as a competing endogenous RNA, promoted caspase-1 expression by targeting miR-214-3p, thereby inducing cardiomyocyte scorching, and thus CACR may be a new therapeutic target for DCM [43]. ELAV-like protein 1 (ELAVL1) maybe plays a critical role in the progression of DCM, Experimental study [44] have found that inhibition of miRNA-9 upregulates ELAVL1 expression and activates caspase-1. Alternatively, treatment with miRNA-9 mimics attenuates hyperglycemia-induced ELAVL1 and inhibits cardiomyocyte pyroptosis. Recent study [45] found that bone morphogenetic protein-2 (BMP-2) was negatively correlated with atrial natriuretic peptide (ANP) and endogenous peptide brain natriuretic peptide (BNP) in serum of patients with type 2 diabetes mellitus combined with chronic heart failure, while in vitro experiments demonstrated that BMP-2 could protect myocardium by inhibition of NLRP3 inflammatory vesicles activation and scorching to protect cardiomyocytes, suggesting that BMP-2 may be a new target for the treatment of DCM. Some scholars further investigated [46] found a novel signaling target in DCM, Nek7/GBP5 pathway activates NLRP3 inflammatory vesicles and disrupts cardiac structure and neovascularization, while BMP-7 inhibits Nek7/GBP5 pathway activation significantly reduces NLRP3 inflammatory vesicle formation, inflammatory cytokines and inflammatory cell infiltration, improving cardiac remodeling and function in DCM. However, the above signaling pathways have been less studied in DCM, and their specific mechanisms need to be further explored. With further understanding of the mechanisms of cardiomyocyte scorching, more new studies are expected to create new therapeutic approaches for patients with DCM.

## Diabetic nephropathy and pyroptosis

DN is a chronic disease that affects approximately 40% of patients with diabetes [47]. It is characterized by thickening of the tubular basement membrane and glomerular membrane, proliferation of thylakoid cells, accumulation of extracellular matrix and progressive thylakoid hypertrophy [48]. The most critical pathological changes are tubulointerstitial inflammation and fibrosis, which are major risk factors for chronic renal failure and end-stage renal disease. [49]. Hyperglycemia induces persistent chronic inflammation and plays a key role in the development and progression of DN [50]; inflammation is involved in the pathogenesis of DN through multiple pro-inflammatory cytokines, including ex vivo monocyte chemotactic protein-1 (MCP-1), tumor necrosis factor-α (TNF-α), IL-6, IL-8, and IL-1β [51, 52]. Persistent hyperglycemia promoting excessive production of ROS is a key factor in NLRP3 inflammatory vesicle activation. The role of NLRP3 inflammatory vesicle activation-induced pyroptosis in DN has now attracted widespread attention. Researchers have observed expression of NLRP3 and caspase-1 in podocytes and endothelial cells of kidneys from patients with diabetic nephropathy and mice [53], and that podocytes are important targets of damage in the early stages of DN. Similarly, it was shown that podocytes stimulated by high homocysteine anemia in [54] or in high-fat diet-induced mouse podocytes, the formation of NLRP3 inflammatory vesicles was closely associated with the development of glomerular injury. In addition, in the stz-treated DN rat model, pyroptosis-related proteins and pro-inflammatory cytokines were increased, and the percentage of scorched cells was increased [55], providing further evidence for the involvement of pyroptosis in DN pathogenesis. In addition to NLRP3 inflammatory vesicle activation associated with DN pathogenesis, Wang et al. [56] reported that NLRC4 inflammatory vesicles activated and secreted inflammatory cytokines under high glucose conditions, leading to scorched death of renal tubular epithelial cells. In addition, non-classical pyroptosis pathways have been shown to be involved in podocyte injury in DN mice. Cheng et al. found that high glucose treatment significantly promoted caspase-11 expression, GSDMD cleavage, and IL-1β release, whereas knockdown of caspase-11 or GSDMD attenuated these changes [57]. One study [58] found that NLRP3 inflammatory vesicles may play a role in high glucose-induced glomerular thylakoid cell activation and inflammation, and that naringin alleviates glomerular thylakoid cell injury by inhibiting NLRP3/caspase-1/ IL-1β signaling pathway-mediated expression of inflammatory factors as a potential new therapy for DN. Sodium–glucose cotransport protein 2 (SGLT2) inhibitors and dipeptidyl peptidase 4 inhibitors (DPP4I) are used to treat type 2 diabetes, and recent studies have reported [59] The combination of SGLT2 inhibitor and DPP4 inhibitor slows the progression of DN by inhibiting NLRP3 inflammatory vesicle activation. lncRNAs, miRNAs and circRNAs regulate the role and potential mechanisms of pyroptosis in DN development. lncRNA-MALAT1 and miR-23c have pro-scorching and anti-scorching properties in DN, respectively. The expression of MALAT1 was significantly increased and the expression of miR-23c was significantly decreased in diabetic rats and high glucose exposed HK2 cells. downregulation of MALAT1 or upregulation of miR-23c expression inhibited HK-2 pyroptosis. further studies revealed that miR-23c, as a target of MALAT1, directly inhibited the expression of ELAVL1 In turn, it decreased the expression of its downstream protein NLRP3, suggesting that MALAT1 is regulating the action of miR-23c on its target gene ELAVL1 to regulate renal tubular epithelial pyroptosis. It provides a new direction for the elucidation of DN pathogenesis and treatment. [10, 60]. Ding et al. [61] found that miR-21-5p in macrophage-derived extracellular vesicles (EVs) affected cell scorch-mediated podocyte injury by increasing ROS production and activating NLRP3 inflammatory vesicles. In addition, circ_WBSCR17 was highly expressed in diabetic mice and high-glucose-treated renal tubular epithelial cells and HK-2 cells, and further studies revealed that circ_WBSCR17 exacerbated the inflammatory response and fibrosis in renal tissues by targeting and regulating the miR-185-5p/SOX6 axis [62]. Cellular scorch plays an important role in renal cell injury and DN pathogenesis, including caspase-1-mediated classical cellular scorch and caspase-4/5/11-mediated non-classical cellular scorch. The NLRP3/caspase-1/GSDMD signaling axis is a key mechanism of cellular scorch in DN progression. Most current studies have been conducted in animal models. Therefore, further exploration of the detailed mechanisms and potential role of pyroptosis in renal tissue biopsies of DN patients is needed.

## Diabetic retinopathy and pyroptosis

DR is a common chronic microvascular complication of diabetes, the pathogenesis of which remains unclear and may be associated with persistent inflammatory damage to the retinal neurovascular unit (NVU) due to a hyperglycemic state, and the chronic inflammatory response induced by pyroptosis may be involved in the pathological changes of the NVU [63]. The retinal NVU is composed of retinal neurons, glial cells, vascular endothelial cells, and pericytes [64]. Previous studies [65] suggest that retinal neuronal degeneration or death is present in the early stages of DR and may precede microvascular lesions. It has been found that retinal ganglion pyroptosis induced by the caspase-8/HIF-1α/NLRP12/NLRP3/NLRC4 pathway under ischemic–hypoxic conditions may be a cause of retinal neuronal degeneration [66, 67], and in addition, previous studies have reported [68], immunohistochemical results showed that NLRP3, ASC, and caspase-1 were specifically localized to the ganglion cell layer of the diabetic rat retina. Müller cells and astrocytes of the retinal NVU are involved in retinal structural support and maintenance of retinal homeostasis. Increased caspase-1 activity in Müller cells under high glucose conditions, increased IL-1β production, and subsequent induction of cellular scorchogenesis [69]. Inhibition of the caspase-1/IL-1β pathway, on the other hand, controls the scorch death of Müller cells [70]. The above evidence suggests that pyroptosis may be an important factor in Müller cell death under high glucose conditions. In addition, Müller pyroptosis leads to neuronal dysfunction and the release of pro-inflammatory factors such as IL-1β leading to endothelial cell death, breaking the integrity of the BRB and inducing cell-free capillary formation, which is one of the major pathological changes in DR [71, 72]. Microglia are retina-specific intrinsic immune cells that monitor the retinal microenvironment and remove metabolic waste products. Retinal ischemia–reperfusion injury is closely associated with DR progression [73], and studies have shown that [74] retinal ischemia–reperfusion injury leads to the occurrence of retinal microglia focal death associated with lncRNA H19. Therefore, microglia scorch death may also be involved in the development of DR. Furthermore, recent studies [75] showed that high sugar upregulated the protein expression of NLPR3, caspase-1, GSDMD, and IL-1β in retinal microglia, suggesting that high sugar induces retinal microglia scorch death through the NLPR3 inflammatory vesicle signaling pathway. Meanwhile, several studies have found that scorch death of retinal vascular endothelial cells and pericytes is involved in the pathological changes of DR. Gan J et al. [76] found that high glucose conditions by NLPR3/caspase-1/NLPR3/caspase-1/GSDMD pathway under scorch death mediated releases large amounts of pro-inflammatory factors leading to peripapillary retinal cell loss. the P2X7/NLRP3 pathway amplifies the inflammatory response through an ATP feedback loop under hyperglycemic and inflammation-inducing conditions, promoting inflammatory responses, pyroptosis, and apoptosis in vascular endothelial cells [77]. And recent studies [78] found that high glucose induces P2X7R activation of the NLPR3 inflammatory vesicle pathway leading to scorching of human retinal microvascular endothelial cells (HRMECs), whereas relaxin-3 inhibits the activation of P2X7R and NLRP3 inflammatory vesicles and delays the progression of DR. Prostaglandin E (an inflammatory mediator) induces HRMECs focal death through upregulation of NLRP3 inflammatory vesicles and inflammatory chemokine expression to promote the development of DR [79]. Recent studies [80] showed that downregulation of lipid transport protein 2 inhibited high glucose-induced caspase-1-mediated pyroptosis in HRMECs and slowed the progression of DR. In recent years, studies on lncRNA s, miRNAs and circRNAs in pyroptosis have provided new targets and ideas for DR prevention and treatment. circFAT1 enhances retinal pigment epithelial cell autophagy and inhibits its pyroptosis by mediating the expression of the m6A reader protein YTHDF2 [81]. Knockdown of CircZNF532 protects retinal pigment epithelial cells from high glucose-induced apoptosis and scorching by regulating the miR-20b-5p/STAT3 axis [82]. miR-192 has been shown to be involved in DR progression by a specific mechanism that inhibits high-glucose-induced scorch death in RPE cells through regulation of the FTO/NLRP3 signaling pathway [83] MiR-200c-3p is highly expressed in high glucose-induced HRMECs, and knockdown of MiR-200c-3p attenuates high glucose-induced scorch death in HRMECs [84], miR-200c-3p may be a potential therapeutic target for DR prevention and treatment. lncRNA myocardial infarction-associated transcript ( MIAT) is thought to be a key regulator of DR microvascular dysfunction, and recent studies [85] found that MIAT promotes caspase-1-dependent pericyte scorching by antagonizing the inhibitory effect of miR-342-3p on its target CASP1, and thus the MIAT/ miR-342-3p /CASP1 pathway may provide a new direction for the mechanism of pericyte loss and the treatment of DR.

## Diabetic peripheral neuropathy and pyroptosis

Diabetic peripheral neuropathy (DPN) is caused by hyperglycemia, which causes oxidative stress and inflammatory responses that damage nerve tissue. Previous studies [86] demonstrated that Chevron's cells have antioxidant and anti-inflammatory neuroprotective effects, and when it becomes dysfunctional it promotes the progression of DPN, Cheng et al. [86] found that strychnine prevented the scorching of RSC96 cells by inhibiting ROS production and NLRP3 inflammatory vesicle activation, suggesting that strychnine inhibited the scorching inflammatory response by antioxidant to delay the progression of DPN.

## Diabetic periodontal disease and pyroptosis

Diabetes mellitus can also cause periodontal homeostasis imbalance and cause dental periodontal disease susceptibility to the disease—diabetic periodontal disease (DP) [87]. Recent studies [88] showed that high glucose activates pyroptosis via the caspase-1/GSDMD/IL-1β pathway and inhibits the proliferation and differentiation of alveolar bone osteoblasts. Zhou et al. [89] found alveolar bone destruction in a mouse model of diabetic periodontitis and increased expression of GSDMD-positive cells and NLRP3 inflammatory vesicles in gingival tissue, which was partially reversed by metformin, suggesting that NLRP3-mediated scorch death has an important role in diabetic periodontitis and that gingival tissue pyroptosis may be a major cause of the inflammatory response, and that metformin treatment may improve local inflammation. In 2021, a study [90] found that hyperglycemia induces macrophage scorch death and leads to impaired cell function and gingival destruction may be involved in the pathogenesis of diabetic periodontal disease, and that GSDMD activation and pyroptosis in the hyperglycemic state is mediated by NLRC4 inflammatory vesicles, then NLRC4 phosphorylation may be a potential therapeutic target to inhibit this process; a contemporaneous study [91] demonstrated that metformin also delayed the damage to periodontal tissue from hyperglycemia-induced macrophage scorching inflammatory response.

## Diabetic keratopathy and pyroptosis

Diabetic keratopathy** (**DK) is also one of the common chronic ocular complications of diabetes mellitus and is often associated with pathological changes such as delayed corneal wound healing, reduced corneal subepithelial nerve density, corneal endothelial damage, and impaired corneal perception [92] More seriously, uncontrolled delayed corneal wound healing may increase susceptibility to bacterial keratitis, corneal ulceration, and even perforation [93]. Under physiological conditions, NLRP3 inflammatory vesicles are necessary for corneal injury repair and nerve regeneration, however, in the case of diabetes, continued activation of NLRP3 inflammatory vesicles leads to delayed corneal wound healing and impaired nerve regeneration. Recent studies [94] suggest that the pathogenesis of DK may be related to the production of accumulated AGEs by ROS promoting excessive activation of NLRP3 inflammatory vesicles. Significantly accelerating diabetic corneal epithelial wound healing and nerve regeneration by genetic and pharmacological blockade of the AGEs/ROS/NLRP3 inflammatory vesicle axis suggests that NLRP3 inflammatory vesicles are expected to be potential targets for DK treatment [94]. Diabetic corneal endotheliopathy is a refractory ocular complication characterized by corneal edema and endothelial loss of compensation, which is a serious threat to vision. Long-stranded non-coding RNA KCNQ1OT1 is associated with the pathophysiological mechanisms of various complications of diabetes [13, 95], recent studies confirm that the KCNQ1OT1/miR-214/caspase-1 signaling pathway may be a novel mechanism for the progression of diabetic corneal endothelial lesions and that KCNQ1OT1 has the potential to be a new therapeutic target [96].

## Diabetes-related cognitive decline and pyroptosis

Diabetes-related cognitive decline (DACD) is an important chronic complication of diabetes, and studies have shown that people with diabetes mellitus have a higher risk of developing Alzheimer's disease (AD) than the general population, especially in Eastern populations [97]. The current findings support that the pathogenesis of DACD is related to a chronic neuroinflammatory response and blood–brain barrier (BBB) disruption due to hyperglycemia [98] related, but the exact mechanism remains unclear. High-mobility group protein b1 (HMGB1) is a ubiquitous nuclear protein that is released extracellularly after cell activation, stress, injury, or apoptosis and plays an important role in neurodegenerative diseases by mediating neuroinflammation [99, 100]; an in vivo trial demonstrated that [101] that inhibition of HMGB1 may alleviate NLRP3 inflammatory vesicle-mediated inflammatory responses in hippocampal neurons via the TLR4/MAPKs/NF-κB signaling pathway. Also, targeting the HMGB1/TLR4 signaling pathway may delay or prevent the increased apoptosis and reduced autophagy of neuronal cells induced by high glucose and hypoxia, thereby improving cognitive dysfunction [102]. It has been suggested that HMGB1 may induce vascular endothelial pyroptosis through activation of NLRP3 inflammatory vesicles leading to BBB dysfunction [103], a recent study found that prickly mangosteen protects against cognitive dysfunction in diabetic mice by inhibiting HMGB1/TLR4/NF-κB signaling and NLRP3 inflammatory vesicle activation and reducing oxidative stress and inflammation [104], the above study provides direct evidence that HMGB1 may be a potential therapeutic target for DACD.

## Conclusion

Only English-language literature from the Web of science core database was included in this study, which may have led to a degree of bias in the search and inclusion of the study results. In addition, although the search strategy used in this study was able to cover the research articles in this field to a great extent, there is still a possibility that the search was not comprehensive. However, by systematically analyzing the literature on diabetes mellitus and pyroptosis in the Web of Science database, this paper presents a multidimensional view of the research results in this field during 2011–2022. The main features of the research in this field and the current new progress and main problems of the research in this field are pointed out, which can provide new ideas and references for the future research layout and research decisions of the research scholars in this field.

Inflammatory pyroptosis is a double-edged sword, and the challenge is whether the key proteins involved in pyroptosis can be used as a clinical biomarker to detect the progression of diabetes mellitus and its various chronic inflammatory complications, and how to safely regulate pyroptosis for the purpose of early prevention and treatment of diabetes mellitus and its complications, which require further research to explore the exact mechanisms of pyroptosis in the progression of diabetes mellitus and its complications. Taken together, research aimed at addressing these issues and innovative technologies related to them will provide new avenues and directions for the prevention and treatment of diabetes mellitus and its complications in the future.

## Data Availability

The original contributions presented in the study are included in the article or supplementary material. Further inquiries can be directed to the first author.
